# Development of the automated temperature control system of the main gas pipeline

**DOI:** 10.1038/s41598-023-29570-4

**Published:** 2023-02-22

**Authors:** Vadim Fetisov, Yury V. Ilyushin, Gennadii G. Vasiliev, Igor A. Leonovich, Johannes Müller, Masoud Riazi, Amir H. Mohammadi

**Affiliations:** 1 Department of Petroleum Engineering, Saint Petersburg Mining University, Saint Petersburg, Russia; 2 Department of System Analysis and Management, Saint Petersburg Mining University, Saint Petersburg, Russia; 3 Department of the Construction and Repair of Gas and Oil Pipelines and Storage Facilities, Gubkin Russian State University of Oil and Gas (National Research University), Moscow, Russia; 4 Department of the Construction and Repair of Gas and Oil Pipelines and Storage Facilities, Gubkin Russian State University of Oil and Gas (National Research University), Moscow, Russia; 5 Leipzig University, 04109 Leipzig, Germany; 6 Shiraz University, Shiraz, Iran; 7 Discipline of Chemical Engineering, School of Engineering, University of KwaZulu-Natal, Howard College Campus, King George V Avenue, Durban, 4041 South Africa

**Keywords:** Chemical engineering, Energy infrastructure, Mechanical engineering

## Abstract

This article presents the results of a numerical experiment and an analysis of temperature fields (coolers for gas) using cooling elements in the case study gas pipeline. An analysis of the temperature fields demonstrated several principles for the formation of a temperature field, which indicates the need to maintain a relative temperature for gas pumping. The essence of the experiment was to install an unlimited number of cooling elements on the gas pipeline. The purpose of this study was to determine at what distance it is possible to install cooling elements for the optimal gas pumping regime, regarding the synthesis of the control law and the determination of the optimal location and assessment of control error depending on the location of the cooling elements. The developed technique allows for the evaluation of the developed control system's regulation error.

## Introduction

In a rapidly growing economy, the issue of providing the necessary number of raw materials to consumers is acute. One of the main sources of raw materials are hydrocarbons, natural gas, and oil. In the course of their processing, various materials and oil products appear to provide the population of the planet with the necessary products of production. With the development of the regions of the Far North (Russia), there is a need to search for technologies for the extraction and transportation over long distances of hydrocarbon raw materials in the presence of various chemical compounds, such as paraffins. The extraction of these types of raw materials is a rather laborious task. Another problem is its subsequent transportation. Raw materials cannot be completely processed on site. Therefore, the crude product enters the field pipeline. The presence of impurities in the raw product has a significant impact on the durability and wear resistance of pipeline transport.

It is also important to note that weather conditions play an important role in the transportation process in the far north. In areas where the average annual temperature can vary from −50 to + 40 degrees Celsius, the pipeline is additionally affected by the physical properties of the metal, whose daily stretching and compression on the pipe walls can lead to deformation and destruction of the entire pipeline.

Unlike oil, if the hydrocarbon feedstock in question is natural gas and the temperature rises, the gas becomes viscous, which makes it difficult to transport it further through the pipeline.

On the one hand, to eliminate these problems, automatic heating (for oil) and maintaining a predetermined temperature (for gas) on the pipeline have been developed. However, such heating of the pipeline is local in nature. Often, a heating element is installed in a certain part of the pipeline, heating it to a high value. And then reheating is applied over a long distance. This method of heating pipelines is not cost-effective because it uses a lot of energy, which drives up the cost of the product.

On the other hand, an alternative application involves laying the pipeline underground. This method is more efficient since the temperature balance is maintained for a longer period. But the implementation of underground laying is not always possible due to areas of permafrost, which leads to further subsidence of the soil. For a long time, the method of laying the heating cable and the method of measuring the thermal impact on the pipeline have been known.

The first study in this direction began with the work of the well-known oil scientist in petroleum geology, Gubkin I. M. In his studies, he described methods of influencing the reservoir and the pump-compressor system to extract extra-viscous oil from the reservoir. Based on his method, scientists around the world have described in their scientific papers’ alternative methods of thermal impact on the pipeline to improve the rheological properties of the extracted raw materials. The author of "Calculation methods and algorithms (pipeline gas transport)," Sardanashvili S.A., described calculation methods and algorithms in a form focused on their practical application in the development and operation of computer systems for the dispatch control of natural gas transport, solving problems of designing and reconstructing gas transmission systems. Lurie M.V. et al., in a study titled "Modeling of Oil Product and Gas Pipeline Transportation," considered the main oil and gas pipelines and methods of thermal influence on them^1^. However, when analyzing the literature, there are no methods for calculating the thermal regime of tank farms and pumping stations.

In other studies, conducted by Lanzano, Erickson, and Nikolaev, the authors analyzed the pipeline infrastructures, presented their calculation methods under various operating conditions, and substantiated the dependences of the hydraulic calculation of oil pipelines transporting high-viscosity oils with complex rheological properties^2–4^. Moreover, Chizhevskaya et al. presented a system of managerial decisions based on the analysis of the work of dispatchers at oil and gas transport facilities. The authors have developed a new technology for monitoring the effectiveness of dispatch control in safety monitoring and methodological support using neural network technologies and machine learning at oil and gas storage facilities^5^. In another study, Zolotov et al. determined a correction factor for converting the ratio of sensor resistance to gas concentration. They developed a program for plotting graphs based on parameters read from sensors for convenient data presentation, and later they developed a program for collecting and storing data from sensors in a file^6^. Moreover, Wu et al.^7^ described field tests and presented numerical simulations using the Timoshenko beam theory and explosion stress wave theory, which take shear effects into account shear effects. Further, the authors of the paper^8–10^ investigated asphaltene deposition in porous media and predicted production profiles based on uncertainty, which improved oil recovery efficiency. Golik et al. presented the author's mathematical model and approval of the methodology for thermal engineering calculations of multilayer oil pipelines. A section of an oil pipeline passing in difficult geocryological conditions is modeled, a method for calculating thermal processes occurring in the "pipe-soil" system is described, and the main results obtained are described^11^. The authors of these works^12–16^ showed the importance of oil and gas temperature control during transportation and storage. In^17–19^, the authors noted that the current composition of raw materials also affects the temperature field. However, these studies were of a local nature in relation to a particular deposit. The systematic nature of the study was first demonstrated in Table 1. This works are showed the possibility of applying the theory of systems with distributed parameters to the analysis of complex multi-parameter systems.

**Table 1:** The applying the theory of systems with distributed parameters.

Author	References
Based on the obtained approximation model, a synthesis of the system for the hydrolytosphere processes parameters control was implemented	Pershin et al.^20^
In this paper a present a mathematical model of the hydrolytospheric process, verify the mathematical model	Pershin et al.^21^
Methods to construct control algorithms for various modifications of control actions are proposed. The results obtained are illustrated by examples of synthesizing automatic control systems of nonstationary heat conduction processes	Rapoport et al.^22^

The papers^23,24^ describe the tasks of ensuring the safe operation of oil and gas pipelines. In the other studies conducted by scholars, they analyzed the need to develop a thermal field control system in various operating conditions and pipeline operating conditions^25–33^. Thus, in works^34,35^ the authors analyzed the existing problem of pipeline systems safety. Which is based on a combination of Bayesian network construction and Dempster-Scheifer evidence theory, which is an alternative method of accident assessment on trunk pipelines, and the proposed structure can provide a more realistic analysis of the consequences of accidents, because it can consider the conditional dependence in the accident process. In the article^36^, the authors attempt to verify whether the method can be used the Discriminant Analysis and Classification (DAC) to achieve the aforementioned goals and to predict the future behavior of network pipes. As case studies, the authors used three pipeline networks that transported different types of fluids (oil, gas and water). For each investigated network, the DAC method was used to classify the pipes into two groups (failed/successful) based on simple variables (pipe/network characteristics) and dimensionless connection variables, and several scenarios were analyzed. In^37^, the authors analyzed the problem of safe operation of gas pipelines under pressure. The results in the work showed that the accident scenario should be considered as a constraint for determining the safe distances in the vicinity of natural and petroleum gas pipelines. The results were further processed to obtain functional diagrams for the rapid assessment of accidents. The authors^38^ in their work investigate how interdependencies between different factors can affect the results of the analysis. This research aims to help owners of transportation and distribution pipeline companies in risk management and decision making to consider the multivariate consequences that can result from pipeline failures. In^39^, a Bayesian belief network (BBN) probabilistic approach to internal corrosion hazard assessment for oil and gas pipelines is presented. The developed BBN model can identify vulnerable sections of the pipeline and rank them accordingly to improve the efficiency of informed decision making. In a study^40^, the authors developed mathematical models that predict the cause of oil pipeline failure based on factors other than corrosion. Regression analysis and artificial neural network models have been developed based on historical pipeline accident data. With these models, operators can make decisions based on predictions of expected failure causes and take necessary actions to prevent accidents.

The works^41–43^ also speak about this, the study of which prompted the authors of this article to conduct a complex analysis and develop a numerical method for calculating the temperature field for efficient and safe operation. So in works^44,45^ are described researches of the analysis of work of gas pipelines by means of a method of a fuzzy complex estimation at construction of mathematical dependences. They are reflected in^46,47^. The authors of works^48,49^ on the basis of the thermal–hydraulic analysis and identification of patterns in the work of gas pipelines conducted an analysis of the cloud theory in building a model of gas pipelines.

Table 2 presents the work of the authors who studied the problem of hydrocarbon transport using the apparatus of distributed systems.

**Table 2:** Presents the research of distributed systems.

Author	References
The presents a study of the temperature distribution as a distributed heating circuit's action result and process of initial heating function formation influenced by the action of uniformly distributed sources is also investigated	Martirosyan et al.^50^
The formula for determining the time of maximum temperature value reaching in the middle of the segment exposing to a source at an arbitrary point of the segment was proposed	Martirosyan et al.^51^
This paper is present a mathematical and computational framework based on h, p, k and variationally consistent integral form is utilized a finite element computational process for two dimensional steady, isothermal as well as non-isothermal fluid flows for power law and Carreau models of viscosity	Surana et al.^52^
An Adaptive Method of Lines algorithm is formulated for the solution of Euler system of equations, which fully simulates slow and fast transients and present two test cases present the improvement of the numerical solution from grid adaptation	Tentis et al.^53^
The method allows for continual updating of occurrence probabilities for adverse events and failure probabilities of safety barriers for successive real time data from industry	Baksh et al.^54^
In this paper are analyzed how to apply cross-impact modeling for described aims the collaborative development of scenarios out of large event sets	Bañuls et al.^55^

When writing this article the studies related to the operation of offshore pipelines have been analyzed. In works^56–58^ the authors offer and show the model of marine pipeline operation, risks arising at ultrahigh pressure, and also ecological consequences at accidents. The works^32,59,60^, which describe the monitoring of gas and oil pipelines operation, are devoted to the ecological problem. In works^61–63^ investigations describing the work of gas networks in an emergency situation are presented, the correlation of accidents to assess leaks is developed. These researches are continued by the authors of^64–66^, who investigate qualitative and quantitative methods of risk assessment of gas pipelines operation at changing their operation modes. Investigations of works^67,68^ show the economic component of calculations of gas pipeline operation, advantage of natural gas supply by pipelines over LNG supply, as well as calculation of gas leakages at accidents and estimation of this value, which finally influences the price of natural gas. The works^69–71^ introduce the reader to the modeling of transient processes in natural gas pipelines. The authors of the works presented numerical simulation and simulation results in software simulators. These studies are interesting because they show the reader the comparison of results and their practical application on real pipeline systems. Similar studies are reflected in papers^72–74^, in which the authors presented methods of spectral element least squares for nonlinear hyperbolic differential equations in the study of gas flow through pipelines, as well as the appearance of the dispersed phase in the gas flow. The authors of^75–77^, introduce readers to the spectral least-squares method for two-dimensional Maxwell equations, Navier–Stokes equations, and research the development of an underground pipeline monitoring system.

The authors of Seung-Mok Shin et al.^78^, developed a real-time monitoring system for detecting extraneous damage on a gas pipeline. For this purpose, a wireless data transmission method was used and the detection locations were limited by the circumstances and the cost of installing sensors. The authors developed calculation and monitoring software using an algorithm using acoustic wave propagation velocity and a database system based on wireless communication and DSP systems. In the works of Surana et al., and Cheng et al., ^79,80^, a spatiotemporal finite element formulation of one-dimensional unsteady Navier–Stokes equations for compressible flow in an Eulerian reference frame for high-speed gas dynamics was developed to ensure the safe operation of gas pipelines for natural gas transportation. Authors Yuhua et al., Francis et al., Girgin et al., analyzed the performance of fluid transport pipelines in estimating failure probabilities using Bayesian belief networks and a fuzzy failure tree^81–83^. At the conclusion of the literature review, we analyzed the works of authors Guo et al., Han and Weng, Hossain and Muromachi^84–86^ who demonstrate a comprehensive risk assessment of trunk pipelines using the fuzzy Petri net model, an integrated method based on Bayesian network. In works^87,88^ authors demonstrate the method of quantitative risk assessment, which is based on the difference of grid sections of the pipeline and review the occurrence of corrosion on the sections of underground gas and oil pipelines during the transportation of oil and gas.

All the works examined demonstrated the significance of developing our own system for controlling the pipeline temperature field with varying regime changes and different types of hydrocarbon feedstock, both for crude oil and natural gas.

It is important to understand that the temperature field propagates in the pipeline along its entire length, taking the thickness of the pipeline into account. Therefore, in the mathematical model, it is necessary to consider the spatial distribution along the entire pipeline as well as the thermal effect on it.

In this study, using heating elements (heaters) that perform the function of a continuous heating element that forms a thermal field at all points of the pipeline is proposed. However, this does not lead to the overheating of some sections of the pipeline, and it is proposed to replace these heating elements with more economical and practical ones, such as impulse and sectional ones, which will ultimately have an economic and practical effect. The installation of such heating elements will help maintain a constant temperature during oil transportation and a constant temperature during gas transportation.

Therefore, a problem statement for the development of a spatially distributed control system for the temperature field of a pipeline transporting hydrocarbon raw materials is generated, and based on the Fourier series and Green's function, a mathematical dependence is presented.

The article continues in "The mathematical model" Section with a brief review of the mathematical model, a numerical example of a problem statement with initial and boundary conditions. In addition, two simplified models are derived from the original one. "Numerical resolution" Section shows the numerical solution of the performance modeling method and the system formulation proposed for integrating these models, along with the mesh to be used for the computer implementation in each case. "Results" Section presents the results of the study, and finally, the findings of the study are presented in "Conclusions" Section.

## The mathematical model

### Initial and boundary conditions

**Figure 1:**
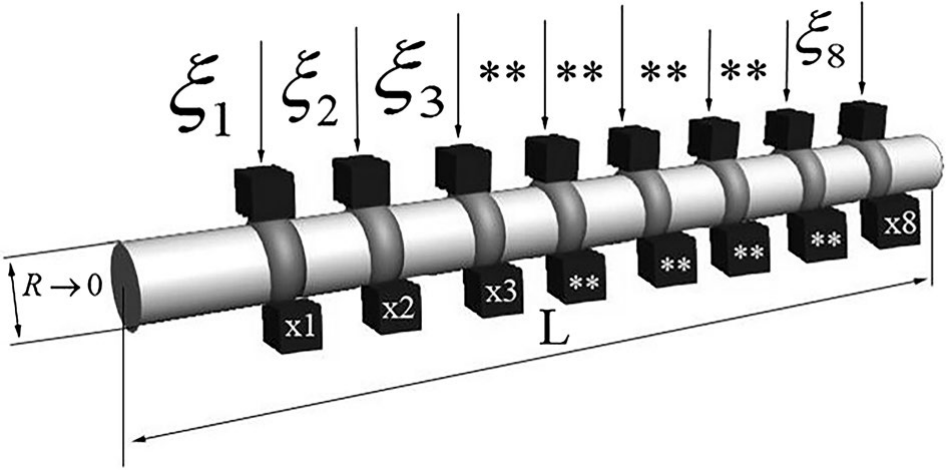
Schematic representation of the pipeline.

Consider a pipeline with an inner radius *R* and length *L* made of a material characterized by the thermal diffusivity of the material *a*^*2*^. Since the raw material in the pipeline is in contact with the inner wall of the pipeline, transferring its heat to it, the temperature of the pipeline wall will be equal to the temperature of the raw material. Thus, the diameter of the pipeline can be considered tending to zero, but not equal to zero. Thus, the pipeline can be represented graphically in Fig. 1.

*R* is the inner radius of a pipe; $\xi$ is point (coordinate along the *X* axis) of the location of the heating element;$x$ is point (coordinated along the X axis) of the location of the temperature sensor; and L is pipeline length.

#### The working principle of the sectional gas coolers

In static mode, the gas pipeline with pulse section coolers is not connected to an electrical supply. In this mode, no electric current is supplied to the coolers and no temperature field is generated. All structural elements located on the metal pipe are at rest at the temperature of the transported medium. The result of compressing gas at compressor stations is an increase in its temperature at the outlet of the compressor station. The initial value of the gas temperature and the pressure volumes in the pipeline determine the value of the gas temperature. Too high a gas temperature at the outlet of the compressor station can cause negative consequences: destruction of the insulating coating of the pipeline and also lead to high stresses in the pipe wall. However, excessive reduction of process gas pressure leads to increased energy consumption for gas compression (due to increased gas consumption).

In cold climates, in areas with frozen ground, measures to cool the gas to sub-zero temperatures are important. This is necessary to prevent the formation of melted soils around the pipeline walls, as this ground penetration can lead to displacement of the pipeline and cause an accident. If the gas is not cooled, it will start to expand, it will become more viscous. Additional energy will be needed to transport it. In dynamic mode, the gas pipeline on which the sectional coolers with the refrigerant are installed is connected to the power grid. In this mode, a pulse current is applied to the X1,X2,X3…X8 cooling elements. Their temperature decreases. Over time they begin to reduce the temperature of the pipe section and the pipeline as a whole. The gas continues to be transported without additional energy and there is no influence of the temperature field on the ground. In comparison to soil stabilizers that are used today the temperature formation field as well as the energy costs are reduced. It is important to note that the number and length of installation of such heaters is not limited.

The cooling elements and sensors will be placed in series to ensure efficiency. Once the system has been designed, it is obvious that having so many cooling elements is not practical for maintaining temperature. Thus, in this study we will obtain a method for determining the optimum (smallest) number of gas cooling elements that will provide the required temperature maintenance. This will be done by keeping *T(x,t)* within *Tza*d. In this case, the initial temperature field of the pipeline *φ(x)*, expressed as the input impact *U* at point *x* at time *t*, will be described by the following expression:

1 $$\begin{gathered} \frac{\partial U}{{\partial t}} = a^{2} \left( {\frac{{\partial^{2} U}}{{\partial x^{2} }}} \right), - \infty < x < \infty ,t > 0 \hfill \\ U(x,0) = \varphi (x), - \infty < x < \infty , \hfill \\ \end{gathered}$$

where $\partial U$ is an input action *U* at point *x*; $\partial t$ is time; $\varphi (x)$ is the initial temperature field of the pipeline.

And:

2 $$\begin{gathered} \hat{O} (p) = \frac{1}{{\sqrt {2\pi } }}\int\limits_{ - \infty }^{\infty } {\varphi (y)e^{ - ipy} dy} \hfill \\ U(p,0) = C = \hat{O} (p) \hfill \\ U(p,t) = \hat{O} (p)e^{{ - a^{2} p^{2} i}} \hfill \\ \end{gathered}$$

We can conclude that:

$$U(x,t) = \frac{1}{2\pi }\int\limits_{ - \infty }^{\infty } {e^{{ - a^{2} p^{2} i}} (} \int\limits_{ - \infty }^{\infty } {\varphi (y)e^{ - ipy} dy)e^{ - ipx} dp} = \frac{1}{2\pi }\int\limits_{ - \infty }^{\infty } {\varphi (y)(} \int\limits_{ - \infty }^{\infty } {e^{ - ipy} e^{{ - a^{2} + ipx}} dp)dy}$$

3 $$\begin{gathered} ( - a^{2} p^{2} - 2\frac{ipy}{2} + \frac{{(y - x)^{2} }}{{4a^{2} t}}) - \frac{{(y - x)^{2} }}{{4a^{2} t}} - ( - a^{2} p^{2} t - 2\frac{ip(y - x)}{2} + \frac{{i^{2} (y - x)^{2} }}{{4a^{2} t}}) - \frac{{(y - x)^{2} }}{{4a^{2} t}} - \hfill \\ - (a\sqrt {tp} + \frac{{i^{{}} (y - x)}}{2a\sqrt t })^{2} - \frac{{(y - x)^{2} }}{{4a^{2} t}}; \hfill \\ \end{gathered}$$

4 $$\begin{gathered} \int\limits_{ - \infty }^{\infty } {e^{{ - (a\sqrt {tp + } \frac{{i^{{}} (y - x)}}{2a\sqrt t })^{2} - \frac{{(y - x)^{2} }}{{4a^{2} t}})}} dp} = \frac{{e^{{ - \frac{{(y - x)^{2} }}{{4a^{2} t}}}} }}{a\sqrt t }\int\limits_{ - \infty }^{\infty } {e^{{ - U^{2} }} dU = \frac{\sqrt \pi }{{a\sqrt t }}e^{{ - \frac{{y^{2} }}{{4a^{2} t}}}} } \hfill \\ U = a\sqrt {tp} + \frac{iy}{{2a\sqrt t }} \hfill \\ dU = a\sqrt t dp \hfill \\ \end{gathered}$$

5 $$U(x,y) = \frac{1}{{2a\sqrt {t\pi } }}\int\limits_{ - \infty }^{\infty } {\varphi (y)e^{{ - \frac{{(y - x)^{2} }}{{4a^{2} t}}}} dy}$$

6 $$G(y,x,t) = \frac{1}{{2a\sqrt {t\pi } }}e^{{ - \frac{{(y - x)^{2} }}{{4a^{2} t}}}}$$

where G is the discrete values of dimensional balance at point y, *x,* *t.*

The resulting function allows you to determine the value of the temperature field in the pipeline at a fixed point in time without considering the diameter of the pipeline. To account for the diameter, consider a mathematical model of the following form:

7 $$T(0,r,t) = T(l,r,t) = 0$$

where T is the temperature at point *0, l, r,* at time *t.*

8 $$T(x,R,t) = u(x,t)$$

9 $$\frac{\partial T(x,0,\tau )}{{\partial r}} = 0$$

10 $$\frac{\partial T}{{\partial t}} = a^{2} \left( {\frac{{\partial^{2} T}}{{\partial x^{2} }} + \frac{1}{r}\frac{\partial T}{{\partial r}} + \frac{{\partial^{2} T}}{{\partial r^{2} }}} \right)$$

11 $$0 < r < R;\;0 < x < l$$

By applying similar transformations, we obtain a formula for calculating the temperature at a pipeline point:

12 $$T(x,t,\xi ,\tau ) = \frac{2}{l}\sum\limits_{n = 1}^{\infty } {\exp \left[ { - \left( {\frac{\pi na}{l}} \right)^{2} \left( {t - \tau } \right)} \right]\sin \frac{\pi n}{l}} x\sin \frac{\pi n}{l}\xi$$

where *n* is the Fourier series term number; *l* is rod length; *t* is time; *x* is point (coordinate along the *X* axis) of the location of the temperature sensor; *ξ* is the point (coordinate along the *X* axis) of the location of the cooling element; *τ* is moment of switching on the point source; and *a*^*2*^ is given coefficient of thermal diffusivity of the material of the control object.

It is critical to understand that the formed temperature field does not remain constant over time. To consider the dynamic characteristics of the measured point, it is necessary to consider the previously formed impulse.

### Partial differential equations

The pulse of each cooling element will affect neighboring cooling elements and sensors. The effect of the first temperature pulse on the following, say, three cooling elements will be expressed as:

13 $$T(x_{1} ,t,\tau_{0} ,\xi_{1} ) = \frac{2}{l}\sum\limits_{n = 1}^{k} {\exp \left[ { - \left( {\frac{\pi na}{l}} \right)^{2} t} \right]} \sin \frac{\pi n}{l}x_{1} \sin \frac{\pi n}{l}\xi_{1}$$

14 $$T(x_{1} ,t,\tau_{0} ,\xi_{2} ) = \frac{2}{l}\sum\limits_{n = 1}^{k} {\exp \left[ { - \left( {\frac{\pi na}{l}} \right)^{2} t} \right]} \sin \frac{\pi n}{l}x_{1} \sin \frac{\pi n}{l}\xi_{2}$$

15 $$T(x_{1} ,t,\tau_{0} ,\xi_{3} ) = \frac{2}{l}\sum\limits_{n = 1}^{k} {\exp \left[ { - \left( {\frac{\pi na}{l}} \right)^{2} t} \right]} \sin \frac{\pi n}{l}x_{1} \sin \frac{\pi n}{l}\xi_{3}$$

And the influence of the specified impulse on the sensors is expressed as:

16 $$T(x_{1} ,t,\tau_{0} ) = \sum\limits_{i = 1}^{3} {} \sum\limits_{n = 1}^{k} {\frac{2}{l}\exp \left[ { - \left( {\frac{\pi na}{l}} \right)^{2} t} \right]} \sin \frac{\pi n}{l}x_{1} \sin \frac{\pi n}{l}\xi_{i}$$

17 $$T(x_{2} ,t,\tau_{0} ) = \sum\limits_{i = 1}^{3} {} \sum\limits_{n = 1}^{k} {\frac{2}{l}\exp \left[ { - \left( {\frac{\pi na}{l}} \right)^{2} t} \right]} \sin \frac{\pi n}{l}x_{2} \sin \frac{\pi n}{l}\xi_{i}$$

18 $$T(x_{3} ,t,\tau_{0} ) = \sum\limits_{i = 1}^{3} {} \sum\limits_{n = 1}^{k} {\frac{2}{l}\exp \left[ { - \left( {\frac{\pi na}{l}} \right)^{2} t} \right]} \sin \frac{\pi n}{l}x_{3} \sin \frac{\pi n}{l}\xi_{i}$$

or:

19 $$T(x_{j} ,t,\tau_{0} ) = \sum\limits_{i = 1}^{d} {} \sum\limits_{n = 1}^{k} {\frac{2}{l}\exp \left[ { - \left( {\frac{\pi na}{l}} \right)^{2} t} \right]} \sin \frac{\pi n}{l}x_{j} \sin \frac{\pi n}{l}\xi_{i}$$

where $$j = 1,2,...d$$.

The dependence describes the influence of the first impact on each subsequent one, considering the current state:

$$T(x_{1} ,t) = \sum\limits_{i = 1}^{3} {} \sum\limits_{n = 1}^{k} {\frac{2}{l}\exp \left[ { - \left( {\frac{\pi na}{l}} \right)^{2} t} \right]} \sin \frac{\pi n}{l}x_{1} \sin \frac{\pi n}{l}\xi_{i} +$$

20 $$\sum\limits_{n = 1}^{k} \frac{2}{l} \exp \left[ { - \left( {\frac{\pi na}{l}} \right)^{2} \left( {t - \tau_{1} } \right)} \right]\sin \frac{\pi n}{l}x_{1} \sin \frac{\pi n}{l}\xi_{1}$$

At the initial stage, the maximum specified power quickly cools the homogeneous object, and the temperature is at its maximum value. However, over time, the temperature reaches *T* = *const* at *t* = *τi*. At this moment, the regulator is activated, which turns on the cooling element ξi and raises the temperature to the set mode necessary to maintain the natural gas temperature. In this case, the location of the cooling element will correspond to the coordinates of the sensor *ξi*. Let us express *ξi* at time *t* = *τi.*

21 $$\begin{gathered} T(x_{2} ,t) = \sum\limits_{i = 1}^{3} {} \sum\limits_{n = 1}^{k} {\frac{2}{l}\exp \left[ { - \left( {\frac{\pi na}{l}} \right)^{2} t} \right]} \sin \frac{\pi n}{l}x_{2} \sin \frac{\pi n}{l}\xi_{i} + \hfill \\ \sum\limits_{n = 1}^{k} \frac{2}{l} \exp \left[ { - \left( {\frac{\pi na}{l}} \right)^{2} \left( {t - \tau_{1} } \right)} \right]\sin \frac{\pi n}{l}x_{2} \sin \frac{\pi n}{l}\xi_{1} \hfill \\ \end{gathered}$$

22 $$\begin{gathered} T(x_{3} ,t) = \sum\limits_{i = 1}^{3} {} \sum\limits_{n = 1}^{k} {\frac{2}{l}\exp \left[ { - \left( {\frac{\pi na}{l}} \right)^{2} t} \right]} \sin \frac{\pi n}{l}x_{3} \sin \frac{\pi n}{l}\xi_{i} + \hfill \\ \sum\limits_{n = 1}^{k} \frac{2}{l} \exp \left[ { - \left( {\frac{\pi na}{l}} \right)^{2} \left( {t - \tau_{1} } \right)} \right]\sin \frac{\pi n}{l}x_{3} \sin \frac{\pi n}{l}\xi_{1} \hfill \\ \end{gathered}$$

or:

23 $$\begin{gathered} T(x_{1} ,t) = \sum\limits_{i = 1}^{3} {} \sum\limits_{n = 1}^{k} {\frac{2}{l}\exp \left[ { - \left( {\frac{\pi na}{l}} \right)^{2} t} \right]} \sin \frac{\pi n}{l}x_{1} \sin \frac{\pi n}{l}\xi_{i} + \hfill \\ \sum\limits_{n = 1}^{k} \frac{2}{l} \exp \left[ { - \left( {\frac{\pi na}{l}} \right)^{2} \left( {t - \tau_{1} } \right)} \right]\sin \frac{\pi n}{l}x_{1} \sin \frac{\pi n}{l}\xi_{1} + \sum\limits_{n = 1}^{k} \frac{2}{l} \exp \left[ { - \left( {\frac{\pi na}{l}} \right)^{2} \left( {t - \tau_{2} } \right)} \right]\sin \frac{\pi n}{l}x_{1} \sin \frac{\pi n}{l}\xi_{3} \hfill \\ \end{gathered}$$

24 $$\begin{gathered} T(x_{2} ,t) = \sum\limits_{i = 1}^{3} {} \sum\limits_{n = 1}^{k} {\frac{2}{l}\exp \left[ { - \left( {\frac{\pi na}{l}} \right)^{2} t} \right]} \sin \frac{\pi n}{l}x_{2} \sin \frac{\pi n}{l}\xi_{i} + \hfill \\ \sum\limits_{n = 1}^{k} \frac{2}{l} \exp \left[ { - \left( {\frac{\pi na}{l}} \right)^{2} \left( {t - \tau_{1} } \right)} \right]\sin \frac{\pi n}{l}x_{2} \sin \frac{\pi n}{l}\xi_{1} + \sum\limits_{n = 1}^{k} \frac{2}{l} \exp \left[ { - \left( {\frac{\pi na}{l}} \right)^{2} \left( {t - \tau_{2} } \right)} \right]\sin \frac{\pi n}{l}x_{2} \sin \frac{\pi n}{l}\xi_{3} \hfill \\ \end{gathered}$$

25 $$\begin{gathered} T(x_{3} ,t) = \sum\limits_{i = 1}^{3} {} \sum\limits_{n = 1}^{k} {\frac{2}{l}\exp \left[ { - \left( {\frac{\pi na}{l}} \right)^{2} t} \right]} \sin \frac{\pi n}{l}x_{3} \sin \frac{\pi n}{l}\xi_{i} + \hfill \\ \sum\limits_{n = 1}^{k} \frac{2}{l} \exp \left[ { - \left( {\frac{\pi na}{l}} \right)^{2} \left( {t - \tau_{1} } \right)} \right]\sin \frac{\pi n}{l}x_{3} \sin \frac{\pi n}{l}\xi_{1} + \sum\limits_{n = 1}^{k} \frac{2}{l} \exp \left[ { - \left( {\frac{\pi na}{l}} \right)^{2} \left( {t - \tau_{2} } \right)} \right]\sin \frac{\pi n}{l}x_{3} \sin \frac{\pi n}{l}\xi_{3} \hfill \\ \end{gathered}$$

Or in a general view:

26 $$\begin{aligned} T(x,t) &= \sum\limits_{i = 1}^{d} {} \sum\limits_{n = 1}^{k} {\frac{2}{l}\exp \left[ { - \left( {\frac{\pi na}{l}} \right)^{2} t} \right]} \sin \frac{\pi n}{l}x\sin \frac{\pi n}{l}\xi_{i} = \frac{2}{l}\left[ {\left( {\exp \left[ { - \left( {\frac{\pi a}{l}} \right)^{2} t} \right]\sin \frac{\pi }{l}x\sin \frac{\pi }{l}\xi_{1} }\right.}\right. \\&\quad \left. {\left. {+ + \exp \left[ { - \left( {\frac{2\pi a}{l}} \right)^{2} t} \right]\sin \frac{2\pi }{l}x\sin \frac{2\pi }{l}\xi_{1} + \cdots + \exp \left[ { - \left( {\frac{k\pi a}{l}} \right)^{2} t} \right]\sin \frac{k\pi }{l}x\sin \frac{k\pi }{l}\xi_{1} ) + } \right)} \right. \hfill \\ & \quad + \left( {\begin{array}{*{20}c} {} \\ {} \\ \end{array} } \right.\exp \left[ { - \left( {\frac{\pi a}{l}} \right)^{2} t} \right]\sin \frac{\pi }{l}x\sin \frac{\pi }{l}\xi_{2} + \exp \left[ { - \left( {\frac{2\pi a}{l}} \right)^{2} t} \right]\sin \frac{2\pi }{l}x\sin \frac{2\pi }{l}\xi_{2} \\&\quad + \cdots + \exp \left[ { - \left( {\frac{k\pi a}{l}} \right)^{2} t} \right]\sin \frac{k\pi }{l}x\sin \frac{k\pi }{l}\xi_{2} \left. {\begin{array}{*{20}c} {} \\ {} \\ \end{array} } \right) + \left. {\left( { - \exp \left[ { - \left( {\frac{\pi a}{l}} \right)^{2} t} \right]\sin \frac{\pi }{l}x\sin \frac{\pi }{l}\xi_{3}}\right.}\right. \hfill \\&\quad \left.{\left.{ + \exp \left[ { - \left( {\frac{2\pi a}{l}} \right)^{2} t} \right]\sin \frac{2\pi }{l}x\sin \frac{2\pi }{l}\xi_{3} + \cdots + \exp \left[ { - \left( {\frac{k\pi a}{l}} \right)^{2} t} \right.\left. {\sin \frac{k\pi }{l}} \right]x\sin \frac{k\pi }{l}\xi_{3} } \right) + \cdots } \right] \hfill \\ & = \left. { \frac{2}{l}\left[ {\exp \left[ { - \left( {\frac{\pi a}{l}} \right)^{2} t} \right]} \right.\sin \frac{\pi }{l}x\left( {\sin \frac{\pi }{l}\xi_{1} + \sin \frac{\pi }{l}\xi_{2} + \sin \frac{\pi }{l}\xi_{3} + ...} \right) + \cdots } \right) \\ & \quad + \exp \left[ { - \left( {\frac{2\pi a}{l}} \right)^{2} t} \right]\sin \frac{2\pi }{l}x\left( {\sin \frac{2\pi }{l}\xi_{1} + \sin \frac{2\pi }{l}\xi_{2} + \sin \frac{2\pi }{l}\xi_{3} + ...} \right) \hfill \\ & \quad + \left. { + \exp \left[ { - \left( {\frac{3\pi a}{l}} \right)^{2} t} \right]\sin \frac{3\pi }{l}x\left( {\sin \frac{3\pi }{l}\xi_{1} + \sin \frac{3\pi }{l}\xi_{2} + \sin \frac{3\pi }{l}\xi_{3} + ...} \right) + ...} \right] \hfill \\ \end{aligned}$$

By considering the power factor of the cooling element and expanding it into a Fourier series, then we get:

27 $$S_{1} = \int\limits_{0}^{l} {\sin \frac{\pi }{l}} xdx = - \frac{l}{\pi }\cos \frac{\pi }{l}x\left| {\begin{array}{*{20}c} l \\ {} \\ 0 \\ \end{array} } \right. = - \frac{l}{\pi }\cos \pi + \frac{l}{\pi }\cos 0 = \frac{2l}{\pi }$$

28 $$S_{2} = \int\limits_{0}^{{{\raise0.7ex\hbox{$l$} \!\mathord{\left/ {\vphantom {l 3}}\right.\kern-0pt} \!\lower0.7ex\hbox{$3$}}}} {\sin \frac{3\pi }{l}} xdx = - \frac{l}{3\pi }\cos \frac{3\pi }{l}x\left| {\begin{array}{*{20}c} {\tfrac{l}{3}} \\ {} \\ 0 \\ \end{array} } \right. = - \frac{l}{3\pi }\cos \pi + \frac{l}{3\pi }\cos 0 = \frac{2l}{{3\pi }} = \frac{1}{3}\left( {\frac{2l}{\pi }} \right)$$

29 $$S_{3} = \int\limits_{0}^{{{\raise0.7ex\hbox{$l$} \!\mathord{\left/ {\vphantom {l 5}}\right.\kern-0pt} \!\lower0.7ex\hbox{$5$}}}} {\sin \frac{5\pi }{l}} xdx = - \frac{l}{5\pi }\cos \frac{5\pi }{l}x\left| {\begin{array}{*{20}c} {{\raise0.7ex\hbox{$l$} \!\mathord{\left/ {\vphantom {l 5}}\right.\kern-0pt} \!\lower0.7ex\hbox{$5$}}} \\ {} \\ 0 \\ \end{array} } \right. = - \frac{l}{5\pi }\cos \pi + \frac{l}{5\pi }\cos 0 = \frac{2l}{{5\pi }} = \frac{1}{5}\left( {\frac{2l}{\pi }} \right)$$

$$S_{n} = \int\limits_{0}^{{{\raise0.7ex\hbox{$l$} \!\mathord{\left/ {\vphantom {l n}}\right.\kern-0pt} \!\lower0.7ex\hbox{$n$}}}} {\sin \frac{\pi n}{l}} xdx = \frac{1}{n}\left( {\frac{2l}{\pi }} \right)$$

where $n -$ odd numbers.

So as $$S_{2} = \frac{1}{3}S_{1} ;$$$$S_{3} = \frac{1}{5}S_{1} ;$$$$....;S_{n} = \frac{1}{n}S_{1}$$,

then:

30 $$\begin{gathered} T\left( {\frac{l}{2};0} \right) = \frac{2}{l}\left[ {\sum\limits_{i = 1}^{d} {\sin \frac{\pi }{l}\xi_{i} - \sum\limits_{i = 1}^{{{\raise0.7ex\hbox{$d$} \!\mathord{\left/ {\vphantom {d 3}}\right.\kern-0pt} \!\lower0.7ex\hbox{$3$}}}} {\sin \frac{3\pi }{l}\xi_{i} + \sum\limits_{i = 1}^{{{\raise0.7ex\hbox{$d$} \!\mathord{\left/ {\vphantom {d 5}}\right.\kern-0pt} \!\lower0.7ex\hbox{$5$}}}} {\sin \frac{5\pi }{l}\xi_{i} - ...} } } } \right] = \hfill \\ = \frac{2}{l}\left[ {\sum\limits_{i = 1}^{d} {\sin \frac{\pi }{l}\xi_{i} - \frac{1}{3}\sum\limits_{i = 1}^{d} {\sin \frac{\pi }{l}\xi_{i} + \frac{1}{5}\sum\limits_{i = 1}^{d} {\sin \frac{\pi }{l}\xi_{i} - ...} } } } \right] = \hfill \\ \frac{2}{l}\left( {\sum\limits_{i = 1}^{d} {\sin \frac{\pi }{l}\xi_{i} } } \right)\left( {1 - \frac{1}{3} + \frac{1}{5} - \frac{1}{7} + \frac{1}{9} - ...} \right) \hfill \\ = \frac{2}{l}\sum\limits_{n = 1}^{\infty } {\frac{{\left( { - 1} \right)^{n - 1} }}{2n - 1}} \sum\limits_{i = 1}^{d} {\sin \frac{\pi }{l}} \xi_{i} \hfill \\ \end{gathered}$$

or $$x = \frac{l}{4}$$: $$\sin \frac{\pi }{l}x = \frac{\sqrt 2 }{2}$$, $$\sin \frac{3\pi }{l}x = \frac{\sqrt 2 }{2}$$, $$\sin \frac{5\pi }{l}x = - \frac{\sqrt 2 }{2}$$, …, then

31 $$\begin{gathered} T\left( {\frac{l}{4};0} \right) = \frac{2}{l}\left( {\sum\limits_{i = 1}^{d} {\sin \frac{\pi }{l}\xi_{i} } } \right)\left( {\frac{\sqrt 2 }{2} + \frac{1}{3}\frac{\sqrt 2 }{2} - \frac{1}{5}\frac{\sqrt 2 }{2} - \frac{1}{7}\frac{\sqrt 2 }{2} + ...} \right) = \hfill \\ = \frac{\sqrt 2 }{l}\left( {\sum\limits_{i = 1}^{d} {\sin \frac{\pi }{l}\xi_{i} } } \right)\left( {1 + \frac{1}{3} - \frac{1}{5} - \frac{1}{7} + \frac{1}{9} + ...} \right)_{{}} \hfill \\ \end{gathered}$$

or $$x = \frac{l}{6}$$:$$\sin \frac{\pi }{l}x = \frac{1}{2}$$, $$\sin \frac{3\pi }{l}x = 1$$, $$\sin \frac{5\pi }{l}x = \frac{1}{2}$$, $$\sin \frac{7\pi }{l}x = - \frac{1}{2}$$, $$\sin \frac{9\pi }{l}x = - 1$$,…,

32 $${\text{then}} \quad T\left( {\frac{l}{6};0} \right) = \frac{1}{l}\left( {\sum\limits_{i = 1}^{l} {\sin \frac{\pi }{l}\xi_{i} } } \right)\left( {1 + \frac{2}{3} + \frac{1}{5} - \frac{1}{7} - \frac{2}{9} + ...} \right)$$

As a result:

33 $$\begin{gathered} T(x_{j} ,t) = \sum\limits_{i = 1}^{d} {} \sum\limits_{n = 1}^{k} {\frac{2}{l}\exp \left[ { - \left( {\frac{\pi na}{l}} \right)^{2} t} \right]} \sin \frac{\pi n}{l}x_{j} \sin \frac{\pi n}{l}\xi_{i} + \hfill \\ + \sum\limits_{p} {\sum\limits_{n = 1}^{k} \frac{2}{l} } \exp \left[ { - \left( {\frac{\pi na}{l}} \right)^{2} (t - \tau_{p} )} \right]\sin \frac{\pi n}{l}x_{j} \sin \frac{\pi n}{l}\xi_{z(p)} \hfill \\ \end{gathered}$$

The accuracy of regulation will be determined by the number cooling elements located on the control object. Thus, by setting the maximum possible number of cooling elements, the system will include only the necessary ones. After the system goes into steady state, cooling elements that were not involved will not be needed. Therefore, the number of remaining elements will be the smallest—that is, optimal.

## Numerical resolution

### Method of characteristic simulation

Let us perform modeling of the developed control system for the gas transportation pipeline. Let's say the length of the pipeline section is 10 m. The simulation result is presented in Table 3 and (Supplementary 1 Pipeline temperature field (3D case)).

**Table 3:** Ccalculation results by the number of sensors.

*d* = 9	*d* = 8	*d* = 7	*d* = 6	*d* = 5
tgas[1, 690] = 19	tgas[1, 690] = 19	tgas[1, 690] = 18	tgas[1, 690] = 18	tgas[1, 690] = 48
tgas[2, 690] = 37	tgas[2, 690] = 36	tgas[2, 690] = 31	tgas[2, 690] = 32	tgas[2, 690] = 39
tgas[3, 690] = 49	tgas[3, 690] = 47	tgas[3, 690] = 43	tgas[3, 690] = 37	tgas[3, 690] = 39
tgas[4, 690] = 56	tgas[4, 690] = 51	tgas[4, 690] = 43	tgas[4, 690] = 32	tgas[4, 690] = 38
tgas[5, 690] = 56	tgas[5, 690] = 47	tgas[5, 690] = 34	tgas[5, 690] = 18	tgas[5, 690] = 45
tgas[6, 690] = 49	tgas[6, 690] = 36	tgas[6, 690] = 19	tgas[6, 690] = 26	
tgas[7, 690] = 37	tgas[7, 690] = 19	tgas[7, 690] = 42		
tgas[8, 690] = 19	tgas[8, 690] = 78			
tgas[9, 690] = 14				

*d* = 14	*d* = 13	*d* = 12	*d* = 11	*d* = 10
tgas[1, 690] = 20	tgas[1, 690] = 20	tgas[1, 690] = 19	tgas[1, 690] = 19	tgas[1, 690] = 19
tgas[2, 690] = 39	tgas[2, 690] = 38	tgas[2, 690] = 38	tgas[2, 690] = 38	tgas[2, 690] = 37
tgas[3, 690] = 56	tgas[3, 690] = 55	tgas[3, 690] = 54	tgas[3, 690] = 53	tgas[3, 690] = 51
tgas[4, 690] = 70	tgas[4, 690] = 68	tgas[4, 690] = 66	tgas[4, 690] = 64	tgas[4, 690] = 60
tgas[5, 690] = 80	tgas[5, 690] = 77	tgas[5, 690] = 74	tgas[5, 690] = 69	tgas[5, 690] = 63
tgas[6, 690] = 87	tgas[6, 690] = 82	tgas[6, 690] = 76	tgas[6, 690] = 69	tgas[6, 690] = 60
tgas[7, 690] = 89	tgas[7, 690] = 82	tgas[7, 690] = 74	tgas[7, 690] = 64	tgas[7, 690] = 51
tgas[8, 690] = 87	tgas[8, 690] = 77	tgas[8, 690] = 66	tgas[8, 690] = 53	tgas[8, 690] = 37
tgas[9, 690] = 80	tgas[9, 690] = 68	tgas[9, 690] = 54	tgas[9, 690] = 38	tgas[9, 690] = 19
tgas[1690] = 70	tgas[1690] = 55	tgas[1690] = 38	tgas[1690] = 19	tgas[1690] = 50
tgas[11, 690] = 56	tgas[11, 690] = 38	tgas[11, 690] = 19	tgas[11, 690] = 85	
tgas[12, 690] = 39	tgas[12, 690] = 20	tgas[12, 690] = 21		
tgas[13, 690] = 20	tgas[13, 690] = 56			
tgas[14, 690] = 92				

Where *d* is the number of input elements (coolers) for gas.

As can be seen from the data in the table, sector peaks are observed. This indicates the possibility of turning off some cooling elements. Calculate the place and time of attraction of the cooling elements. In a similar manner, we obtain a two-dimensional equation.

34 $$\begin{gathered} T(x_{j} ,y_{j} ,t) = \sum\limits_{i = 1}^{d} {} \sum\limits_{k,m = 1}^{\infty } {\frac{4}{{l_{1} \cdot l_{2} }}\exp \left[ { - a^{2} \pi^{2} \cdot t \cdot \left( {\frac{{k^{2} }}{{l_{1}^{2} }} + \frac{{m^{2} }}{{l_{2}^{2} }}} \right)} \right] \cdot \sin \left( {\frac{{k \cdot \pi \cdot x_{j} }}{{l_{1} }}} \right)} \times \hfill \\ \times \sin \left( {\frac{{k \cdot \pi \cdot \rho_{i} }}{{l_{1} }}} \right) \cdot \cdot \sin \left( {\frac{{m \cdot \pi \cdot y_{j} }}{{l_{2} }}} \right) \cdot \sin \left( {\frac{{m \cdot \pi \cdot \nu_{i} }}{{l_{2} }}} \right) + \sum\limits_{p} {} \sum\limits_{k,m = 1}^{\infty } {\frac{4}{{l_{1} \cdot l_{2} }} \times } \hfill \\ \times \exp \left[ { - a^{2} \pi^{2} \cdot (t - \tau_{p} ) \cdot \left( {\frac{{k^{2} }}{{l_{1}^{2} }} + \frac{{m^{2} }}{{l_{2}^{2} }}} \right)} \right]\sin \left( {\frac{{m \cdot \pi \cdot y_{j} }}{{l_{2} }}} \right) \cdot \sin \left( {\frac{{k \cdot \pi \cdot x_{j} }}{{l_{1} }}} \right) \times \hfill \\ \times \sin \left( {\frac{{k \cdot \pi \cdot \rho_{z(p)} }}{{l_{1} }}} \right) \cdot \sin \left( {\frac{{m \cdot \pi \cdot \nu_{z(p)} }}{{l_{2} }}} \right); \hfill \\ \end{gathered}$$

From which we express the coordinates of the location of the included cooling element.

35 $$x = \arcsin \frac{{\frac{4}{{l_{1} \cdot l_{2} }} \cdot \sum\limits_{k,m = 1}^{\infty } {} \sin \left( {\frac{m \cdot \pi \cdot y}{{l_{2} }}} \right) \cdot \sin \left( {\frac{k \cdot \pi \cdot \rho }{{l_{1} }}} \right) \cdot \sin \left( {\frac{m \cdot \pi \cdot \nu }{{l_{2} }}} \right) \times \exp \left[ { - a^{2} \pi^{2} \cdot t \cdot \left( {\frac{{k^{2} }}{{l_{1}^{2} }} + \frac{{m^{2} }}{{l_{2}^{2} }}} \right)} \right]}}{G(x,y,\rho ,\nu ,t)} \cdot (\frac{{l_{1} }}{k \cdot \pi })$$

36 $$\begin{gathered} y = \hfill \\ = \arcsin \frac{{\frac{4}{{l_{1} \cdot l_{2} }} \cdot \sum\limits_{k,m = 1}^{\infty } {} \sin \left( {\frac{m \cdot \pi \cdot x}{{l_{1} }}} \right) \cdot \sin \left( {\frac{k \cdot \pi \cdot \rho }{{l_{1} }}} \right) \cdot \sin \left( {\frac{m \cdot \pi \cdot \nu }{{l_{2} }}} \right) \times \exp \left[ { - a^{2} \pi^{2} \cdot t \cdot \left( {\frac{{k^{2} }}{{l_{1}^{2} }} + \frac{{m^{2} }}{{l_{2}^{2} }}} \right)} \right]}}{G(x,y,\rho ,\nu ,t)} \cdot (\frac{{l_{2} }}{m \cdot \pi }) \hfill \\ \end{gathered}$$

Let`s conduct an experimental study under the same conditions. The result is presented in the form of Table 4.

**Table 4:** Turning on the cooling elements.

*l* = *5*	*l* = *6*	*l* = *7*	*l* = *8*
Cooler number = 4	Cooler number = 1	Cooler number = 1	Cooler number = 1
Cooler number = 3	Cooler number = 8	Cooler number = 6	Cooler number = 9
Cooler number = 2	Cooler number = 6	Cooler number = 5	Cooler number = 6
Cooler number = 2	Cooler number = 6	Cooler number = 4	Cooler number = 4
Cooler number = 2	Cooler number = 6	Cooler number = 4	Cooler number = 4
Cooler number = 3	Cooler number = 6	Cooler number = 4	Cooler number = 4
Cooler number = 4	Cooler number = 9	Cooler number = 5	Cooler number = 5
		Cooler number = 1	Cooler number = 2
		Cooler number = 1	Cooler number = 1
		Cooler number = 1	Cooler number = 1

### System formulation

Based on the data obtained, the following conclusion can be drawn: While maintaining the temperature in the pipeline at 25 degrees in a 10-m-long section, only some heaters were activated by the control system. In the experiment where five heaters were installed, only coolers numbers 2, 3, and 4 were used. In the system where six heaters were installed, only coolers numbers 1, 8, 6, and 9 were used. Thus, the economic feasibility of the developed technique was experimentally proven (Supplementary 2 Program 42 cooler sections).

The essence of the experiment was to install an unlimited number of cooling elements on the object under study, which can also play the role of cooling elements if they are used on a gas pipeline under the conditions of synthesis of the control law and determining the optimal location of cooling elements. Building a system is possible if unused elements are removed. The automation scheme built based on this methodology must comply with the qualitative and quantitative characteristics required for control systems. To check the quality of this system, several methods have been developed:

1. Technique for finding the optimal location of impulse cooling or cooling elements in composite control objects. This technique allows us to determine the optimal discretization step for composite and multilayer control objects.
2. Evaluation of the control error depending on the location of the heating or cooling element using the developed technique, which allows for the evaluation of the developed control system's regulation error.

## Results

The purpose of the research work was to conduct a generalised literature review on the problem of loss of gas temperature during long-distance transportation, availability of a control system for pulse cooling of natural gas flow by installing cooling sensors on gas pipelines.

Analysis of the data showed that to date there is no proposed method for calculating heat and energy losses along the length of the pipeline, as well as technologies that could be applied to maintain the temperature regime of natural gas during long-distance transportation. The authors developed a system of heating and cooling elements aimed at improving transportation of the gas medium without loss of gas temperature along the length of the pipeline to prevent hydrate formation, as well as to prevent gas expansion that would complicate its transportation. The authors carried out an analysis of the dynamic temperature fields generated by pulse section heaters. They presented a synthesis of the temperature field control system based on the Green's function of the wall of a multi-section heater-cooler, taking into account the spatial configuration of the pipe. They presented one-, two- and three-dimensional analytical models of controllable temperature field with pulse heating elements, which are distinguished by the use of Green's function in order to accelerate the processes in comparison with finite-difference models. This model is characterised by a hierarchical structure, a reasonable choice of input, internal, measurable and controllable quantities, which makes it possible to develop a mathematical model of the controlled spatial heating process.

## Conclusions

This study presents the results of a numerical experiment and an analysis of temperature fields (coolers for gas) using cooling elements in the case study gas pipeline. An analysis of the temperature fields demonstrated several principles for the formation of a temperature field, which indicates the need to maintain a relative temperature for gas pumping. The essence of the experiment was to install an unlimited number of cooling elements on the gas pipeline. The purpose of this study was to determine at what distance it is possible to install cooling elements for the optimal gas pumping regime, regarding the synthesis of the control law and the determination of the optimal location and assessment of control error depending on the location of the cooling elements. The developed technique allows for the evaluation of the developed control system's regulation error. The developed technique includes the ability to estimate the error given by the coordinates of the cooling elements' locations and the fact that their locations differ. The main results of the study include as below:

1. A mathematical model of the pipeline has been obtained, which makes it possible to determine the temperature field of the pipe at any time, considering the dynamically changing state.
2. A technique has been obtained for determining the installation location of cooling elements that makes it possible to calculate the installation locations for cooling elements, taking the specified temperature regime into account.
3. To maintain the set temperature in the gas pipeline at 25 degrees in a section of 10 km, only 4 out of 6 cooling elements were activated by the control system. Therefore, the economic feasibility of the developed method for determining the optimal temperature for transporting natural gas through the main pipeline was experimentally proven.

The obtained results were validated using various piping schemes. To improve the quality of the functioning of this system, it would be useful to determine the turn-on time of the cooling elements. This will significantly reduce the time for overshooting the pipeline system and save energy in the operation of the compressor station. However, this is the subject of further research.

## Supplementary Information

Supplementary Information 1.

Supplementary Information 2.

Supplementary Information 3.

Supplementary Information 4.

Supplementary Information 5.

## Data Availability

All data generated or analyzed during this study are included in this published article and its Supplementary information files. Request for more details to the corresponding author.
