# Supplementary material for: Development of the automated temperature control system of the main gas pipeline
Source: Sci Rep. 2023 Feb 22;13:3092. doi: 10.1038/s41598-023-29570-4 (PMC9946939; doi:10.1038/s41598-023-29570-4)
Supplement: Supplementary file 2 — Supplementary Information 2. [file 41598_2023_29570_MOESM2_ESM.docx]

The results obtained have been tested on pipeline layouts. As part of the study, it was proposed to replace the existing coolers elements with impulse sectional ones.

A mathematical model of the pipeline has been obtained, which makes it possible to determine the temperature field of the pipe at any time, taking into account the dynamically changing state.

A method for determining the location of the cooling elements has been obtained, which makes it possible to calculate the location of the installation of the cooling elements, taking into account the specified temperature regime.
